# Therapeutic response to pazopanib: case report and literature review on molecular abnormalities of aggressive prolactinomas

**DOI:** 10.3389/fendo.2023.1195792

**Published:** 2023-07-17

**Authors:** Eduardo J. Medina, Youssef M. Zohdy, Edoardo Porto, Juan M. Revuelta Barbero, David Bray, Justin Maldonado, Alejandra Rodas, Miguel Mayol, Bryan Morales, Stewart Neill, William Read, Gustavo Pradilla, Adriana Ioachimescu, Tomas Garzon-Muvdi

**Affiliations:** ^1^ Department of Neurosurgery, Emory University, Atlanta, GA, United States; ^2^ Department of Neurosurgery, Fondazione IRCCS Istituto Neurologico Carlo Besta, Milan, Italy; ^3^ Department of Otolaryngology, Emory University, Atlanta, GA, United States; ^4^ Department of Pathology, Emory University, Atlanta, GA, United States; ^5^ Department of Oncology, Emory University, Atlanta, GA, United States; ^6^ Department of Endocrinology, Emory University, Atlanta, GA, United States

**Keywords:** aggressive, atypical, prolactinoma pituitary adenomas, pazopanib, molecular biomarkers

## Abstract

**Introduction:**

Aggressive prolactinomas (APRLs) pose a significant clinical challenge due to their high rate of regrowth and potentially life-threatening complications. In this study, we present a case of a patient with an APRL who had a trial of multiple therapeutic modalities with the aim to provide a review of molecular abnormalities and management of APRLs by corroborating our experience with previous literature.

**Methods:**

A total of 268 articles were reviewed and 46 were included. Case reports and series, and studies that investigated the molecular and/or genetic analysis of APRLs were included. Special care was taken to include studies describing prolactinomas that would fall under the APRL subtype according to the European Society of Endocrinology guidelines; however, the author did not label the tumor as “aggressive” or “atypical”. Addiontionally, we present a case report of a 56-year-old man presented with an invasive APRL that was resistant to multiple treatment modalities.

**Results:**

Literature review revealed multiple molecular abnormalities of APRLs including mutations in and/or deregulation of ADAMTS6, MMP-9, PITX1, VEGF, POU6F2, CDKN2A, and Rb genes. Mismatch repair genes, downregulation of microRNAs, and hypermethylation of specific genes including RASSF1A, p27, and MGMT were found to be directly associated with the aggressiveness of prolactinomas. APRL receptor analysis showed that low levels of estrogen receptor (ER) and an increase in somatostatin receptors (SSTR5) and epidermal growth factor receptors (EGFR) were associated with increased invasiveness and higher proliferation activity. Our patient had positive immunohistochemistry staining for PD-L1, MSH2, and MSH6, while microarray analysis revealed mutations in the CDKN2A and POU6F2 genes. Despite undergoing two surgical resections, radiotherapy, and taking dopamine agonists, the tumor continued to progress. The patient was administered pazopanib, which resulted in a positive response and the patient remained progression-free for six months. However, subsequent observations revealed tumor progression. The patient was started on PD-L1 inhibitor pembrolizumab, yet the tumor continued to progress.

**Conclusion:**

APRLs are complex tumors that require a multidisciplinary management approach. Knowledge of the molecular underpinnings of these tumors is critical for understanding their pathogenesis and identifying potential targets for precision medical therapy.

## Introduction

Prolactinomas are the most prevalent subtype of pituitary adenomas (PA), accounting for approximately 50% of all PA cases (1). While generally considered benign, a subset of prolactinomas exhibit atypical characteristics such as rapid growth, invasiveness, and resistance to standard treatment (2). Aggressive prolactinomas (APRLs) pose a significant clinical challenge due to their high rate of regrowth and potentially life-threatening mass effect complications (3, 4). Further, many pituitary carcinomas, although very rare among pituitary tumors, are prolactin-secreting tumors that present as macroadenomas in males. Differentiating between APRL and pituitary carcinoma can be challenging, and the presence of distant metastases is the only distinguishing feature that favors the diagnosis of the latter (2, 4).

The standard first-line treatment for prolactinomas are dopamine agonists (DA) such as cabergoline and bromocriptine, which have proven to be effective in reducing tumor size and controlling prolactin levels in 80-90% of patients (5). In cases where patients have an incomplete response to DA, cannot tolerate the medication, or wish to pursue a definitive cure, surgical resection is a viable option. This approach typically results in normalization of prolactin levels in 75-90% of patients, with low morbidity and minimal risk of mortality (6). However, APRL management can be challenging, as these tumors can be resistant to both DA therapy and surgical removal (7). In these instances, radiotherapy and temozolomide (TMZ) are referred to as the third and fourth lines of therapy, with response rates of 75% and 51% respectively (8–11). For APRL who continue to grow despite all of these therapies, patients undergo reoperation, long-course TMZ, or trial of emerging therapies currently under clinical trial investigation (12).

The complexity of aggressive tumors, such as APRLs, has necessitated the development of new patient-tailored precision therapies. This approach requires a deep understanding of the underlying molecular abnormalities for accurate diagnosis, prognosis, and treatment. Multiple molecular abnormalities have been documented in APRLs, including structural chromosomal abnormalities, genetic mutations, hypermethylation, and surface receptor alterations (13–27). In this study, we present a case of a patient with APRL who harbored multiple molecular abnormalities. Despite multiple therapeutic approaches, including DA, multiple surgical resections, TMZ, tyrosine kinase inhibitor (TKI), and anti-PD-L1 therapy, the tumor progression continued to fluctuate. Our study aims to provide a review of molecular abnormalities and management of APRLs by corroborating our experience with previous literature.

## Methods

**Figure 1:**
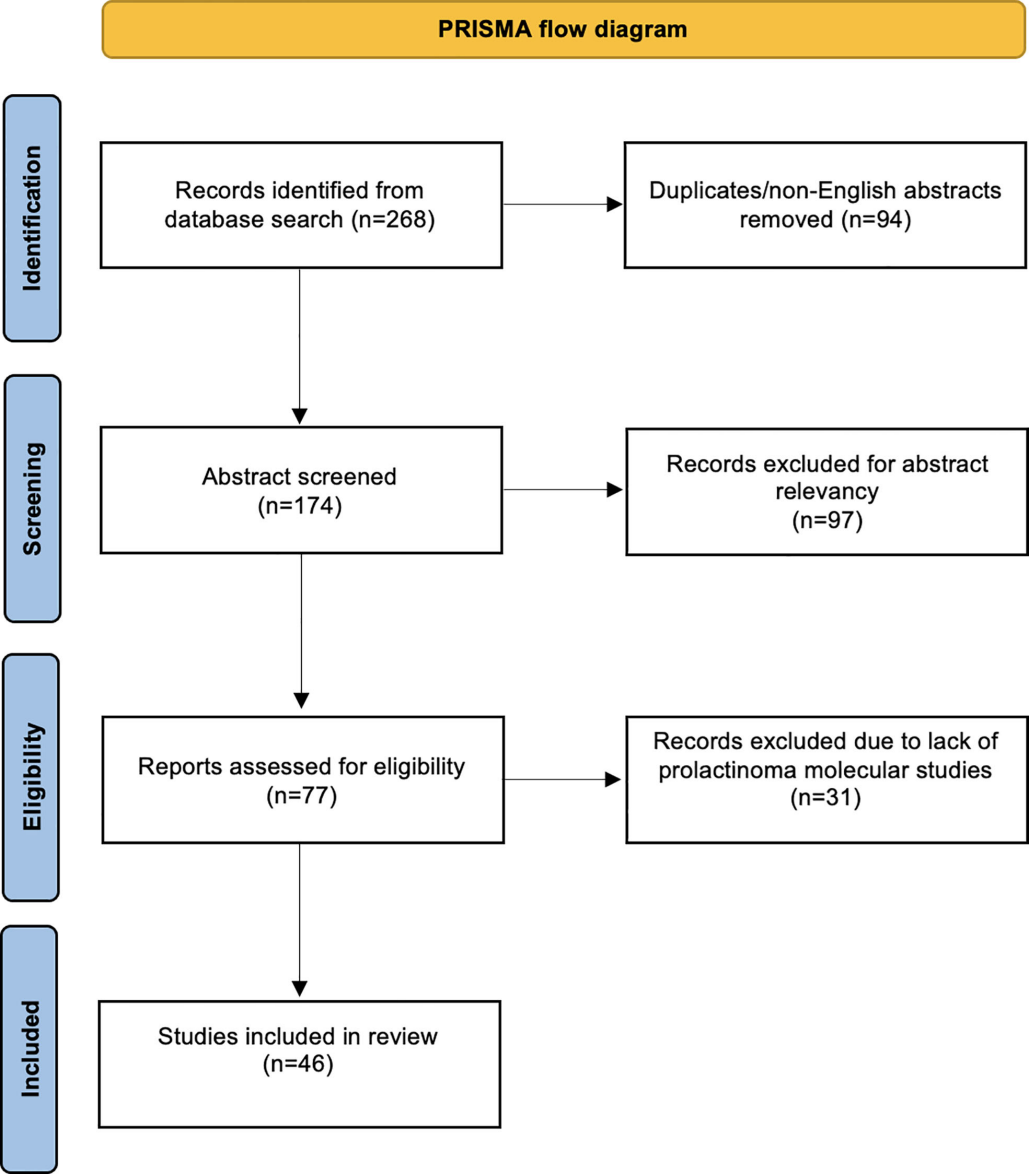
PRISMA flow diagram.

A total of 268 articles were obtained through a literature search conducted using PubMed and Cochrane Central Register of Controlled Trials libraries. The database was searched from inception until 2022. Duplicate and non-English language papers were excluded. Abstracts were screened, and subsequent full-text evaluations were performed for relevant manuscripts. We included case reports and series, and studies that investigated the molecular and/or genetic analysis of APRLs. Studies that lacked molecular characterization of the tumors were excluded. Special care was taken to include studies describing prolactinomas that would fall under the APRL subtype according to the European Society of Endocrinology (ESE) guidelines; however, the author did not label the tumor as “aggressive” or “atypical” (4). Two authors independently screened abstracts and arrived at an agreement. The review was conducted according to the Preferred Reporting Items for Systematic Reviews and Meta-Analyses (PRISMA) guidelines (**Figure 1**) (28).

## Case description

**Figure 2:**
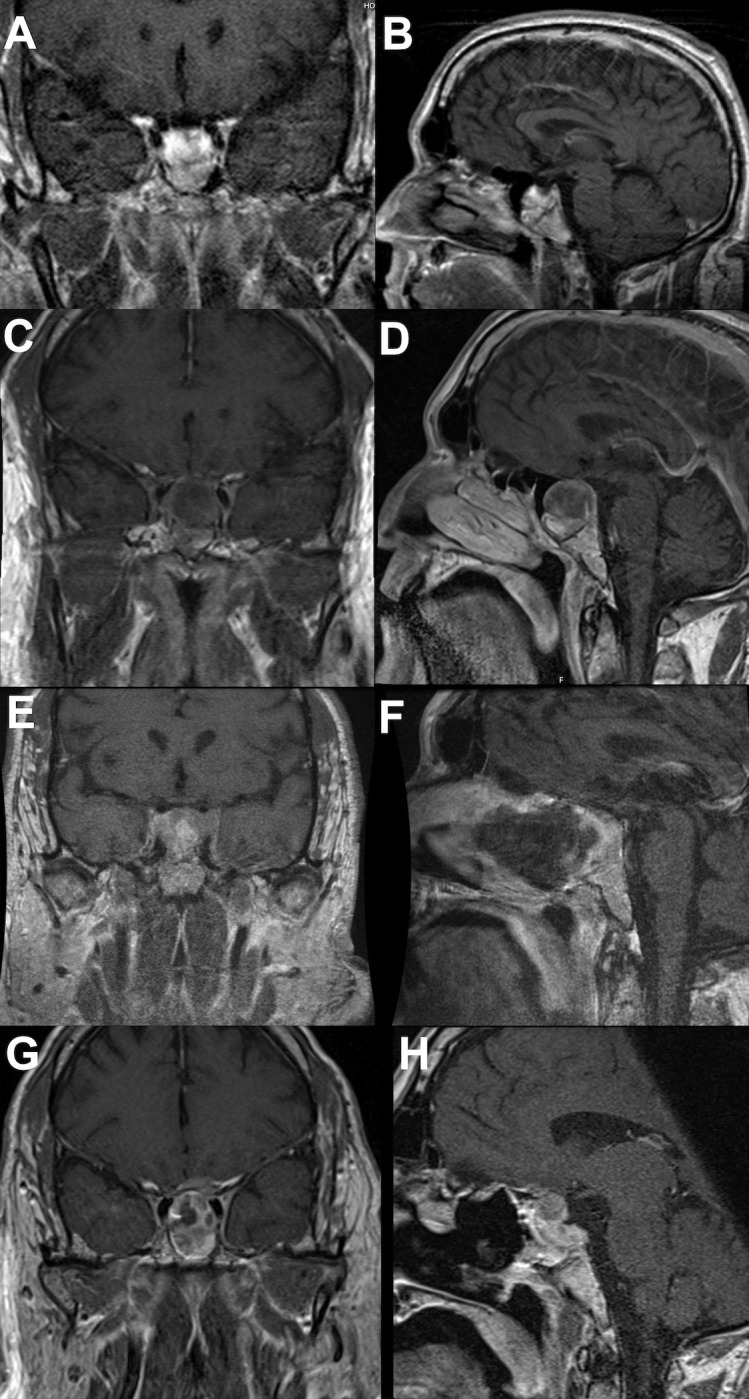
Brain MRI showing tumor progression. (**A** coronal; **B** sagittal) showing initial tumor presentation. (**C** coronal; **D** sagittal) showing tumor prior to first operation. (**E** coronal; **F** sagittal) showing tumor after first operation. (**G** coronal; **H** sagittal) showing continued tumor progression postoperatively and prior to temozolomide therapy.

**Figure 3:**
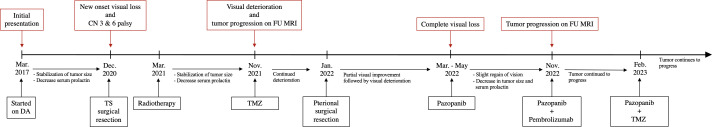
Timeline showing the course of the disease and management. DA, dopamine agonist; CN, cranial nerve; TS, Trans-Sphenoidal; FU, follow-up; TMZ, temozolomide.

A 56-year-old man presented at our institution in March 2017 with altered mental status and diaphoresis, which was believed to be a myocardial infarction. The cardiac workup was negative. However, examination revealed manifestations of hypogonadism and a serum prolactin level of approximately 200 ng/mL. A brain MRI was ordered and revealed a 2 × 2 cm pituitary mass (**Figures 2A, B**). He was diagnosed with a prolactinoma and initiated on cabergoline but experienced gastrointestinal disturbances and was subsequently switched to bromocriptine. The bromocriptine dose was gradually increased to 20mg/day. The tumor size stabilized, and serum prolactin levels decreased (**Figure 3**).

In December 2020, the patient presented with three days of new-onset diplopia, blurry vision in the left eye, and headache. Physical examination showed a left eye adduction deficit with anisocoria, consistent with left cranial nerve (CN) III palsy. The neurological exam also revealed a subtle left CN VI palsy. His serum prolactin level at admission was 330 ng/mL. A brain MRI showed progression of the mass with suprasellar extension, left cavernous sinus invasion, and evidence of apoplexy (**Figures 2C, D**). Surgical resection was recommended, and the tumor was debulked through a transsphenoidal approach (**Figures 2E, F**). Pathological analysis of the resected tissue showed positive immunohistochemical (IHC) staining for prolactin and PIT-1 and a 30% MIB1/Ki-67 proliferation index with increased mitotic activity and nuclear atypia.

Postoperatively, the patient’s CN III palsy improved, and serum prolactin level decreased to 166 ng/mL. The patient was prescribed cabergoline 1 mg twice a week and subsequently underwent adjuvant fractionated radiotherapy (total dose 30 Gy), which was completed 2 months later.

**Figure 4:**
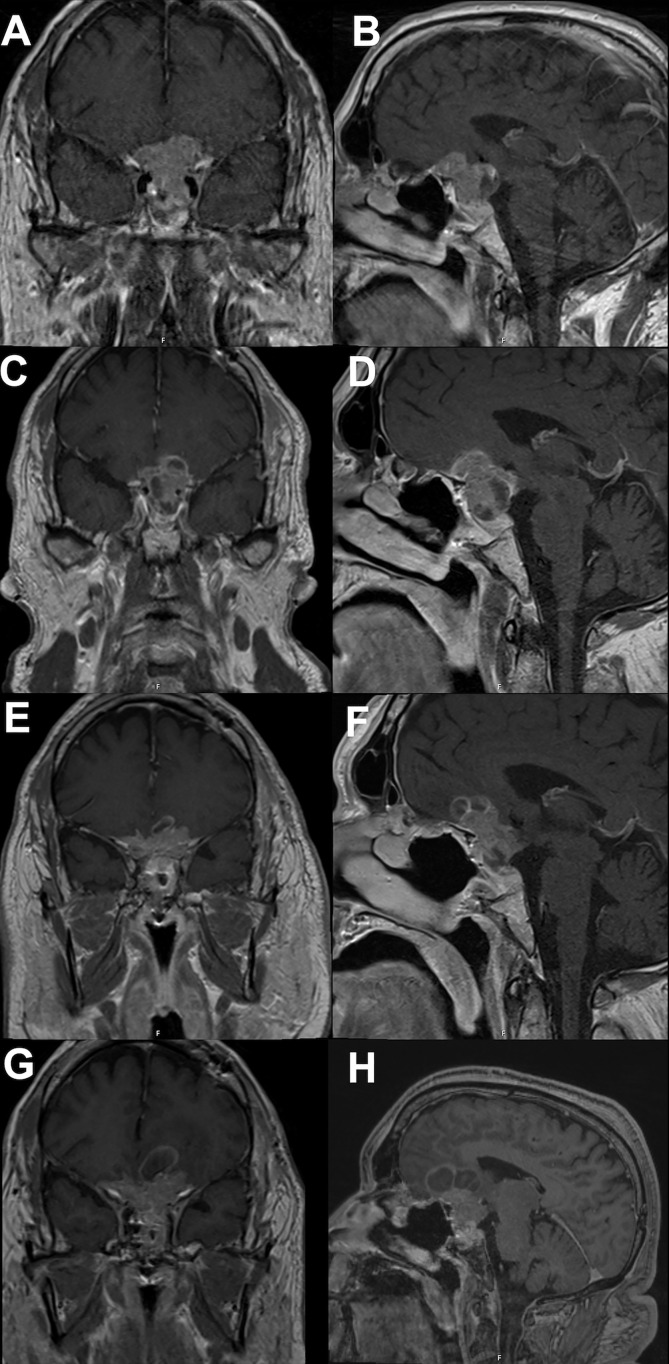
Brain MRI showing tumor progression. (**A** coronal; **B** sagittal) showing continued tumor progression after temozolomide therapy and prior to second operation. (**C** coronal; **D** sagittal) showing tumor prior to pazopanib therapy. (**E** coronal; **F** sagittal) showing tumor response to pazopanib therapy, tumor size slightly decreased and stabilized for 6 months. (**G** coronal; **H** sagittal) showing continued tumor progression despite ongoing therapy.

In November 2021, the patient reported significant decrease in vision of the left eye; follow-up MRI revealed progression of the residual tumor with bilateral invasion of the cavernous sinuses and compression of the left optic nerve (**Figures 2G, H**). The serum prolactin was elevated at approximately 500 ng/mL despite an increase in cabergoline dosage. Clinically, his visual acuity was significantly decreased in both eyes, with only light perception in the left eye. He was prescribed two courses of TMZ; however, his vision deteriorated and the tumor continued to grow (**Figures 4A, B**). In January 2022, he underwent a second operation through a transcranial approach and subtotal resection was achieved, decompressing the optic nerves (**Figures 4C, D**). The resected tumor tissue was sent for detailed histopathological and genetic analysis, and the findings are discussed in the section below. Postoperatively, vision partially improved, but then started deteriorating again two months later to complete blindness. Follow-up MRI revealed progression of tumor size, and the serum prolactin level continued to increase (730 ng/mL).

**Figure 5:**
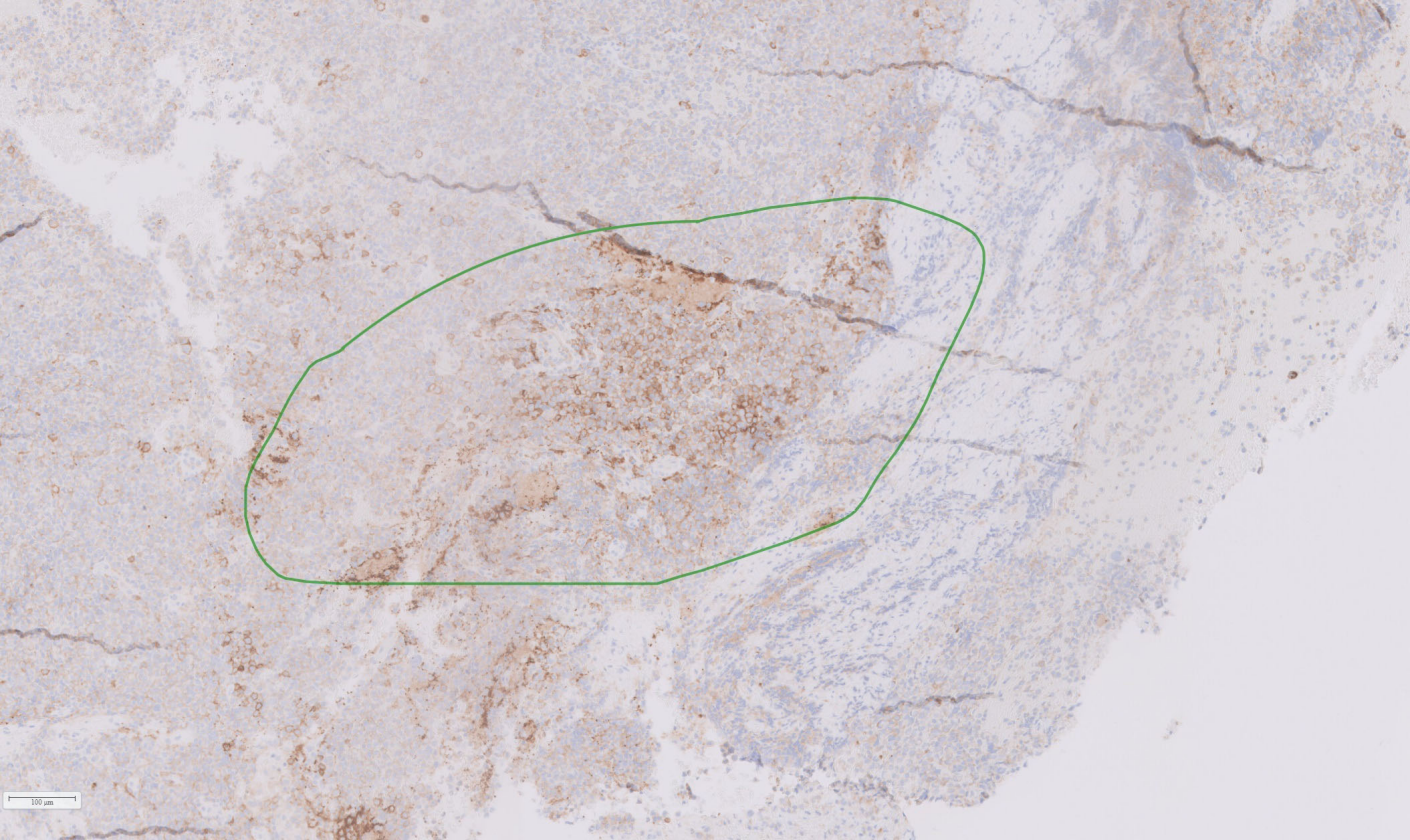
Histological section of resected tumor tissue showing positive PD-L1staining (marked in green).

Based on the tumor aggressiveness and resistance to all standardized lines of therapy, a novel approach was explored. The multi-kinase inhibitor, pazopanib, showed promising results in patients with neuroendocrine carcinomas of gastrointestinal, lung, and pancreatic origins (29). The patient was subsequently started on pazopanib (800 mg daily). His symptoms gradually improved, and after 3 months, there was an improvement in vision in the right eye with partial restoration of visual fields bilaterally. Additionally, serum prolactin decreased to 122 ng/mL, and MRI showed a reduction in tumor size along with a decrease in the cystic and necrotic components of the tumor (**Figures 4E, F**). The patient remained progression-free for six months. However, after 7 months of starting pazopanib treatment, MRI revealed tumor progression (**Figures 4G, H**). Based on PD-L1 positive staining of the tumor tissue (**Figure 5**), the PD-L1 inhibitor pembrolizumab was added to the patient’s treatment regimen, however, the tumor continued to progress. The patient was prescribed a combination therapy of pazopanib and TMZ, yet the tumor still continues to grow.

### Histopathology and molecular analysis

**Figure 6:**
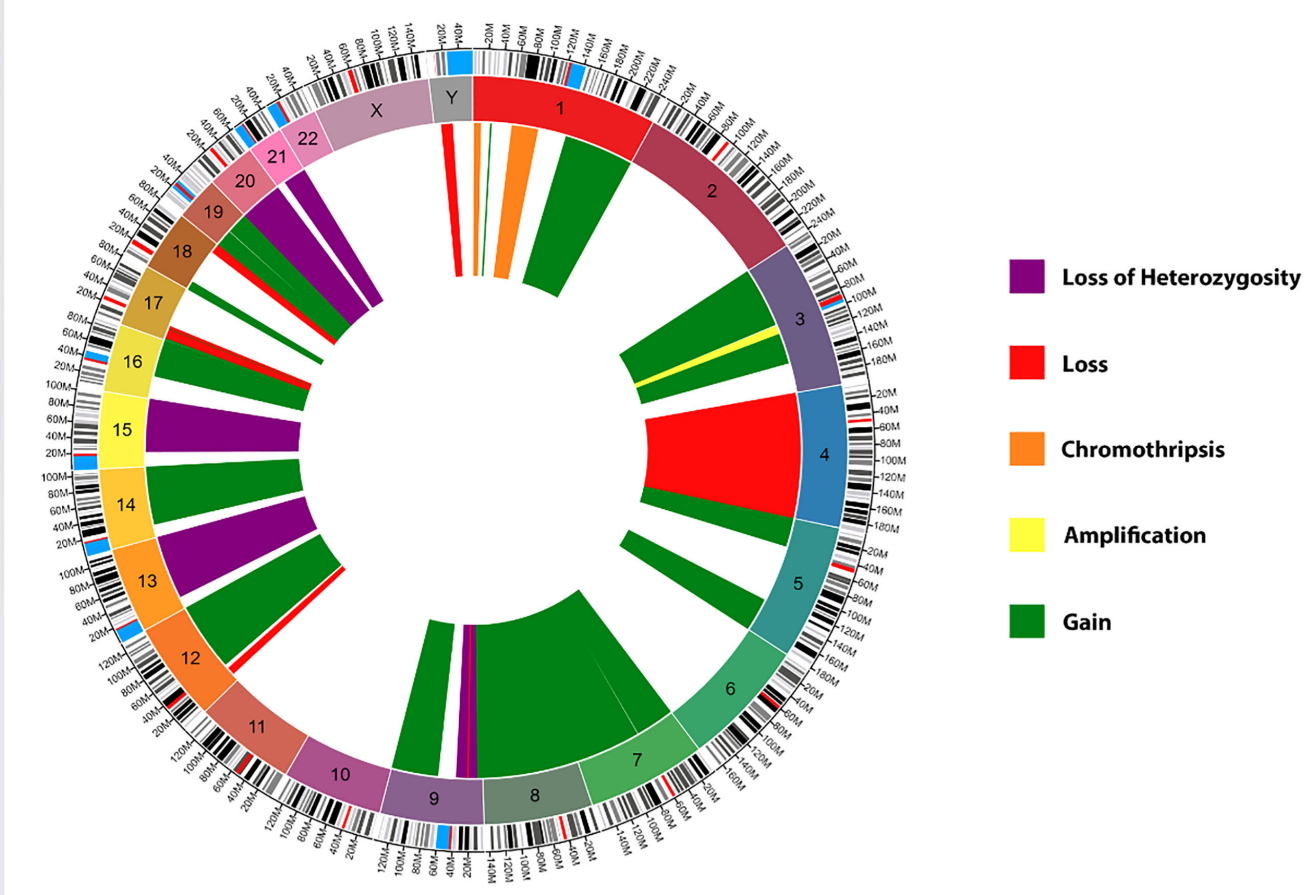
Circos Plot of the genome sequencing by microarray analysis of the patient’s tumor. Green: Chromosomal gain(s), Yellow: Focal chromosome amplification(s), Red: Chromosomal loss(es), Purple: Copy neutral loss(es) of heterozygosity, and Orange: Chromosomal chromothripsis. Created using Circa software by https://omgenomics.com/.

Histopathological examination of the resected tumor tissue revealed pituitary adenoma with foci of necrosis and hemorrhage. IHC staining was positive for PIT-1 and prolactin but negative for FSH, LH, SF1, GH, TSH, ACTH, and TBX19. IHC results were also strongly positive for PD-L1, MLH1, MSH2, MSH6, and PMS2. The MIB1/Ki-67 proliferation index was 30%, mitotic figures were up to 18 per 10 HPF, and nuclear atypia was moderate to severe. Genome sequencing by microarray analysis of the tumor specimen revealed numerous acquired copy number abnormalities, including focal amplification of chromosome 3q, complex aberrations consistent with chromothripsis in two regions of chromosome 1p, regional loss of chromosome 9p (with a portion of focal homozygous loss breaking within the CDKN2A gene), and regional loss of chromosome 17p (containing the TP53 gene). Other abnormalities detected via microarray analysis included regional chromosomal gain(s) in chromosomes 1, 3, 5, 7, 8, 9, 12, 14q, 16, 17p, 18p, and 19; regional chromosomal loss(es) in chromosomes 1p, 4, 9p, 12p, 17p, 18q, and Yq; and copy neutral loss of heterozygosity in chromosomes 8p, 9p, 13q, 15q, 19p, 20, and 21q (**Figure 6**). Genomic DNA sequencing also revealed low-level microsatellite instability, low tumor mutational burden, and low (7%) genomic loss of heterozygosity. HLA sequencing detected A*26:01, A*31:01, B*38:01, B*40:01, C*03:04, and C*12:03 genotypes. Details of the methodology of analysis is present in the supplementary material.

## Discussion

### Pathophysiology

Our presented case highlights the challenges encountered in the treatment of APRL that are resistant to multiple therapeutic modalities. This resistance prompted us to conduct an extensive pathological analysis of the tumor with the goal of identifying specific therapeutic targets. Consistent with previous studies, our analysis revealed molecular abnormalities within the tumor that exhibited variability. IHC results demonstrated positive expression of PD-L1, MSH2, and MSH6. Microarray analysis provided additional insights revealing regional losses in chromosomes 1p and 9p, which included the CDKN2A gene, and loss of heterozygosity on chromosome 13q. Additionally, regional chromosomal gains were observed in chromosome 7, including the POU6F2 gene. These molecular abnormalities have all been previously described in APRL (17, 30, 31).

Throughout literature, most genetic abnormalities encountered in PA patients are associated with syndromic diseases (23). Development of sporadic PAs, which constitute the majority of cases, is attributed to multifactorial tumorigenesis involving genetic disposition, somatic mutations, and endocrine factors (23). The WHO classification of endocrine tumors includes genetic markers for the classification of these neoplasms, such as testing for transcription factor PIT-1, estrogen receptor (ERα), and steroidogenic factor 1 (SF-1) (2). With the increasing incidence of aggressive PAs, such as APRLs, greater emphasis is placed on molecular and genetic profiling since cellular abnormalities serve as potential therapeutic targets for resistant tumors. Therefore, the comprehensive molecular and genetic profiling of PAs, particularly sporadic PAs, is critical for accurate diagnosis, prognosis, and development of targeted therapies (32–34).

Based on broad molecular profiling studies, a total of 726 genes and 13 proteins were found to exhibit different expression patterns in prolactinomas compared to normal tissues (35). However, a subset of molecular abnormalities was specifically associated with APRLs (**Table 1**). These molecular markers include ADAMTS6, MMP-9, TIMP-1, MCM7, ALK7, PTTG1, CUL4A, PITX1, SCN3B, USP8, CRMP1, CCNB1, CENPE, HMGA2, POU6F2, CDKN2A, Galectin-3, DGKZ, VEGF, Rb, and ASK; which were all found to be significantly associated with tumor aggressiveness and invasiveness (24–27, 30, 31, 35–37, 39, 41, 43, 44, 48–50, 53, 54, 58, 65–68). Mismatch repair genes, MSH2 and MSH6, were found to be directly linked to the aggressiveness of both functional and silent prolactinomas (16, 17). Additionally, downregulation of several microRNAs (miR-137, miR-183, miR-15a, and miR-16-1) has been observed in APRLs (15, 56, 59).

**Table 1:** Genetic and Molecular abnormalities of aggressive prolactinomas.

Reference	Genetic, Protein and Receptor abnormalities	Status
Compared with non-aggressive prolactinomas		
Pei et al. (36)	RB	Down-regulated
Bates et al. (37)	11q13, 13q12–14, 10q, and 1p	Down-regulated
Jaffrain et al. (38)	EGF	Up-regulated
Turner et al. (39)	MMP9	Up-regulated
Qian et al. (40)	CDH13 and CDH1	Down-regulated
Wierinckx et al. (24)	PITX1 and SCN3B	Down-regulated
Wierinckx et al. (24)	ADAMTS6, ASK, RACGAP1, CENPE and AURKB, PTTG	Up-regulated
Huang et al. (41)	Galectin-3 and Bcl-2	Up-regulated
McCormack et al. (42)	MGMT	Down-regulated
Qian et al. (43)	HMGA2	Up-regulated
Tanase et al. (44)	PTTG1	Up-regulated
Vlotides et al. (45)	ErbB3	Up-regulated
Cristina et al. (46)	VEGF and CD31	Up-regulated
Miyajima et al. (47)	VEGF	Up-regulated
Raverot et al. (48)	CRMP1, ADAMTS6, CCNB1, CENPE and ASK	Up-regulated
Cooper et al. (21)	EGF and EGFR	Up-regulated
Wierinckx et al. (25)	DGKZ, CD44, TSG101, GTF2H1, HTATIP2	Down-regulated
Righi et al. (49)	Galectin-3	Up-regulated
Sánchez-Ortiga et al. (50)	VEGF	Up-regulated
Arya et al. (51)	MGMT	Down-regulated
Cooper et al. (52)	EGFR, ErbB2, ErbB4, ErbB3	Up-regulated
Roche et al. (15)	miR-183	Down-regulated
Coli et al. (53)	MCM7	Up-regulated
Guo et al. (54)	MMP- 9	Up-regulated
Guo et al. (54)	TIMP-1	Down-regulated
Ma et al. (55)	DNMT1 and DNMT3A	Up-regulated
Uraki et al. (16)	MSH6 and MSH2	Down-regulated
Cooper et al. (20)	EGFR/ErbB2	Up-regulated
Lei et al. (56)	miR-137	Down-regulated
Kara et al. (30)	CDKN2A	Down-regulated
Uraki et al. (17)	MSH6 and MSH2	Down-regulated
Bima et al. (19)	Galectin-3	Up-regulated
Compared with normal pituitary tissue		
Jaquet et al. (57)	SSTR 5	Up-regulated
Finelli et al. (58)	HMGA2	Up-regulated
Bottoni et al. (59)	miR-15a and miR-16-1	Down-regulated
Qian et al. (60)	RASSF1A	Down-regulated
Evans et al. (35)	ADAMTS6 and ADAM28	Up-regulated
Kovacs et al. (61)	MGMT	Down-regulated
Mallea-Gil et al. (62)	VEGF, FGF-2 and CD31	Up-regulated
Fedele et al. (26)	HMGA2	Up-regulated
Principe et al. (27)	ALK7	Up-regulated
Coopmans et al. (63)	SSTR5	Up-regulated
Giuffrida et al. (64)	SSTR2/5	Up-regulated
Miao et al. (31)	POU6F2	Up-regulated
Miao et al. (31)	CYP4B1, DMXL2, EGF, FLT3, MUTYH, RSPH6A and SLCO1B3	NR

NR, Not recorded.

Epigenetic changes have also been implicated in the development of APRLs (23). Specifically, hypermethylation of RASSF1A, Rb1, p27, MEG3, DAPK, GNAS1, MGMT, CDH13, CDH1, and CDKN2A genes has been shown to be more frequent in APRLs (40, 42, 51, 55, 60, 61, 69). This has been attributed to DNA methyltransferases DNTM1 and DNMT3A, whose overexpression has been linked to prolactinoma aggressiveness (23, 55, 70).

Research on APRL receptors has revealed that tumors with low levels of estrogen receptor (ER) exhibit larger size, increased invasiveness, and higher proliferation activity (14, 19, 71). In addition, the presence of somatostatin receptors (mainly SSTR5) and epidermal growth factor receptors (EGFR) have been linked to tumor aggressiveness and resistance to dopamine agonists (DA) (57, 63, 64, 72). Treatment with somatostatin receptor ligands and EGFR tyrosine kinase inhibitors (TKI) have demonstrated therapeutic efficacy in APRL tumors that express SSTR and EGFR, respectively (20, 21, 38, 45, 52, 57, 63–65, 72–75).

### Treatment

APRL typically exhibits a limited response to dopamine agonist therapy, often leading to ineffective results. Additionally, APRL has a tendency to recur even after surgical resection, and it demonstrates resistance to conventional radiotherapy methods. Due to these challenges, the resistance of APRL to standard therapeutic approaches has necessitated the exploration of more targeted treatment strategies based on personalized molecular findings within the tumors.

Temozolomide has shown promise as a pharmacological agent for treating patients with aggressive pituitary tumors, including APRL. Its cytotoxic effects are achieved by inducing methylation of DNA at the O6 position of guanine, which leads to the mispairing of guanine with thymine (61). However, the DNA repair enzyme 6-O-methylguanine-DNA methyltransferase (MGMT) can reverse the effects of temozolomide by removing alkylating adducts and counteracting its action (61). Accordingly, patients with APRL that exhibit low levels of MGMT expression are considered suitable candidates for temozolomide therapy (42). This suggests that MGMT expression, yet not MGMT promoter methylation, can serve as a predictive factor for the response to treatment in aggressive pituitary tumors, including APRL (18). It is important to note that although the correlation between MGMT expression and response to temozolomide has been observed, it has not been definitively confirmed. Therefore, temozolomide has still be utilized as a treatment option for APRL independent of MGMT expression status.

Studies have demonstrated that somatostatin receptor (SSTR) ligands, particularly SSTR5 ligands, exhibit excellent biochemical and tumor responses in the treatment of APRL. These ligands have been associated with tumor shrinkage and rapid antitumor effects. Pasireotide, a multireceptor-targeted somatostatin analog, has shown favorable outcomes in previous studies when used to treat APRLs that express SSTR5 (63, 76). Notably, patients treated with pasireotide experienced progression-free periods up to 7 years (63, 76). Despite these encouraging findings, it is important to acknowledge that comprehensive clinical studies exploring the full potential of SSTR ligands in treating APRL are still lacking.

The exploration of TKIs as a potential therapy for aggressive pituitary tumors, including APRL, has shown promising results. The overexpression of human EGFR2 in some cases of APRL has led to the trial of receptor blockade, which has demonstrated effectiveness (65). In a study evaluating the efficacy of lapatinib, an EGFR/ErbB2 tyrosine kinase inhibitor, in APRL patients, a median decrease of 42% in prolactin levels was observed (20). While in terms of tumor size, after 6 months of lapatinib treatment, three subjects exhibited stable tumor size, one subject experienced a 22% reduction in tumor volume, and two subjects showed tumor growth (20). However, it is important to note that in this particular study, the response to lapatinib was observed irrespective of EGFR/ErbB2 expression (20). This suggests that the effectiveness of lapatinib may not solely depend on the expression levels of these receptors in APRL (20, 52).

Given the relatively high expression of VEGF in aggressive pituitary tumors, anti-VEGF TKIs has been described as a potential treatment option for refractory PAs. Anti-VEGF therapy has been investigated both as monotherapy and in combination with other agents such as temozolomide (TMZ), TMZ and radiotherapy, and pasireotide (22). These approaches hold promise as alternative therapies for refractory PAs that do not respond to conventional treatments. Notably, studies have identified a link between DA resistance and increased VEGF expression (22, 46, 50, 77). In preclinical models, mice with prolactinomas lacking the dopamine 2 receptor (D2R) exhibited higher VEGF levels, which were subsequently reduced with anti-VEGF treatment, leading to decreased tumor size, vascularity, and prolactin levels (78, 79). Similarly, a clinical study reported high VEGF levels in a patient with DA-resistant prolactinoma (62). These findings collectively suggest a potential role for anti-VEGF TKIs therapies in treating DA-resistant prolactinomas.

Multiple anti-VEGF TKI therapies have been described, including bevacizumab and pazopanib. Phase II clinical trials have demonstrated modest clinical activity with bevacizumab in advanced neuroendocrine tumors (80, 81). Pazopanib, on the other hand, is an oral multitargeted TKI that acts through various receptors, including VEGFR types 1–3, fibroblast-derived growth factor receptors (FGFR 1, 3, and 4), platelet-derived growth factor receptors α and β, and stem-cell factor receptor (c-Kit) (82, 83). A systematic review with pooled meta-analysis of Phase II trials of pazopanib in aggressive neuroendocrine neoplasia demonstrated promising results. The rate of stable disease across the trials was 79.6%, with a disease control rate of 90.3% (29). The median progression-free survival was 11.6 months, and the overall survival from all the trials was 24.6 months (29). Pazopanib showed a comparable overall response rate to other TKIs and mTOR inhibitors, with a safety profile similar to drugs in the same class. This suggests that pazopanib may be an effective option for patients with aggressive neuroendocrine neoplasia.

In our patient, the decision to initiate a pazopanib trial was based on previous studies that established a correlation between increased tumor vascularity and intratumoral hemorrhage (which was observed in our patient), with elevated levels of VEGF expression and tyrosine kinase activity (22, 46, 77). Initially, pazopanib demonstrated effectiveness in controlling tumor progression, reducing prolactin secretion, and providing a six-month period without disease progression. However, tumor progression eventually resumed.

The tumor response observed in our patient to pazopanib supports the notion of a favorable therapeutic response in cases of APRL. However, it is important to note that while some cases with aggressive neuroendocrine neoplasia with elevated VEGF levels have shown a positive response to anti-VEGF therapy, a direct correlation between VEGF/VEGFR immunoreactivity and the efficacy of anti-VEGF treatment has not been definitively established (29). Further studies are required to elucidate clear correlations between treatment response and specific response biomarkers in the context of anti-VEGF therapy for aggressive pituitary tumors. Nonetheless, the potential of anti-VEGF therapy in the treatment of APRL is promising.

Finally, immunotherapy has emerged as a potential promising therapy for APRL. The presence of PD-L1 expression, which correlates with high Ki-67 or MIB-1 proliferative index, has prompted the investigation of PD-L1 blockers as a potential treatment option for these tumors. Both preclinical and clinical studies have demonstrated that PD-1/PD-L1 blockade can enhance T cell anti-tumor responses and lead to the expansion of activated CD8+ effector T cells (84). However, the direct impact of PD-L1 blockade on the progression of aggressive pituitary tumors remains a subject of debate (85, 86). While some studies have shown synergistic effects when combining anti-PD-L1 therapy with radiotherapy and/or temozolomide in the treatment of APRL, others, including our case, have observed that PD-L1 blockade failed to halt tumor progression despite PD-L1 positivity (85, 86).

In our presented case, given the failure of multiple therapeutic approaches and the presence of PD-L1 positivity in the patient’s tumor, pembrolizumab was administered as a last resort. However, no noticeable effect was observed in terms of tumor progression or serum prolactin levels. These findings further emphasize the controversial nature of PD-L1 blockade in the treatment of APRL, suggesting that the relationship between PD-L1 expression and the response to PD-L1 blockade is not straightforward. Comprehensive studies are needed to delve into the potential of PD-L1 blockers and other immunotherapies for the treatment of APRL.

## Conclusion

APRLs are complex tumors that require a multidisciplinary management approach. Knowledge of the molecular underpinnings of these tumors is critical for understanding their pathogenesis and identifying potential targets for precision medical therapy. Our patient had partial response to pazopanib. This finding highlights the effect of designing a treatment plan tailored to the molecular abnormalities of tumors and the need for further analysis and documentation of tumor molecular profiling.

## Author contributions

EM, YZ, JM, and AR conducted the literature review. MM, BM, SN, and WR created the figures and molecular analysis and review. EM, YMZ, EP, and JR wrote the first draft of the manuscript. GP, AI, and TG-M reviewed and edited the final draft of the manuscript. All authors contributed to the article and approved the submitted version.
