# Supplementary material for: Therapeutic response to pazopanib: case report and literature review on molecular abnormalities of aggressive prolactinomas
Source: Front Endocrinol (Lausanne). 2023 Jul 17;14:1195792. doi: 10.3389/fendo.2023.1195792 (PMC10388536; doi:10.3389/fendo.2023.1195792)
Supplement: Supplementary file 1 [file DataSheet_1.docx]

Supplementary Material

**Therapeutic Response to Pazopanib: Case Report and Literature Review on Molecular Abnormalities of Aggressive Prolactinomas**

Eduardo J. Medina, BS^1†^, Youssef M. Zohdy, MD^1†^, Edoardo Porto, MD^1,2^, Juan M. Revuelta Barbero, MD, PhD^1^, David Bray, MD^1^, Justin Maldonado, BA^1^, Alejandra Rodas, MD^3^, Miguel Mayol, MD^1^, Bryan Morales, MD^4^, Stewart Neill, MD^4^, William Read, MD^5^, Gustavo Pradilla, MD^1^, Adriana Ioachimescu, MD^6^, Tomas Garzon-Muvdi, MD, MSc^1*^

*** Correspondence:** Corresponding Author: tomas.garzon-muvdi@emory.edu

# Supplementary Data

**Methodology of genetic analysis**

Single Nucleotide Polymorphism Copy Number (SNP-CN) array analysis was performed using the Thermo Fisher Scientific OncoScan FFPE Assay Kit on genomic DNA isolated from formalin-fixed paraffin-embedded (FFPE) tissue (1,2). The OncoScan platform queries 239,038 markers (19,038 non-polymorphic CN markers and 220,000 SNP markers, with increased density within approximately 900 cancer or cancer related genes) and includes detection of copy number abnormalities, gene deletion and amplification events as well as loss of heterozygosity and allelic imbalances across the entire human genome. Data analysis was performed using the Thermo Fisher Scientific CHAS software and OncoScan Nexus Express software and aligned to the National Center for Biotechnology Information (NCBI) human build GRCh38 assembly. The OncoScan assay utilizes molecular inversion probe technology which is optimized for FFPE samples. The assay was performed according to the manufacturer's protocol. All controls performed as expected. SNP-CN array analysis does not detect balanced chromosomal aberrations such as reciprocal translocations, Robertsonian translocations, inversions, balanced insertions; point mutations, epigenetic changes, low-level mosaicism or tumor burden, and imbalances that are not represented on the microarray. Copy number changes are based on relative estimates; therefore, polyploidy cannot be reliably determined. Databases used in the analysis of this specimen included: UCSC Genome Browser (http://genome.ucsc.edu), Database of Genomic Variants (http://dgv.tcag.ca/dgv/app/home), DECIPHER V 6.0 (http://decipher.sanger.ac.uk), NCBI Human Genome Resources (http://www.ncbi.nlm.nih.gov/genome), and OMIM (http://www.ncbi.nlm.nih.gov/omim).

References:

1- Yin D, et al. High resolution genomic copy number profiling of glioblastoma multiforme by single nucleotide polymorphism DNA microarray. Mol Cancer Res. 2009;7(5), 665-77.

2- Dougherty MJ, et al. Technical utilization of high-resolution single nucleotide polymorphism based oligonucleotide arrays in diagnostic studies of pediatric patients with solid tumors. Cancer Genet. 2012;205(1-2):42-54.
